# Influence of Race/Ethnicity and Household Median Income on Penile Cancer Mortality

**DOI:** 10.7759/cureus.40909

**Published:** 2023-06-24

**Authors:** Oluwasegun A Akinyemi, Mojisola E Fasokun, Terhas Asfiha Weldeslase, Oluwatayo Adeoye, Pamela W Coleman

**Affiliations:** 1 Health Policy and Management, University of Maryland School of Public Health, College Park, USA; 2 Surgery, Howard University, Washington, DC, USA; 3 Epidemiology and Public Health, University of Alabama at Birmingham, Birmingham, USA; 4 Surgery, Howard University College of Medicine, Washington, DC, USA; 5 Medicine, St. Elizabeth's Medical Center, Brighton, USA; 6 Surgery, Howard University Hospital, Washington, DC, USA

**Keywords:** survival outcomes, cancer-specific mortality, median household income, race/ethnicity, penile carcinoma

## Abstract

Introduction: Penile cancer, while relatively rare in developed nations, presents substantial disparities in outcomes among different demographic groups. Previous research has shown race/ethnicity and socioeconomic status, often proxied by household median income, to be critical determinants of health outcomes across various diseases.

Objective: This study examines the association of race/ethnicity and household median income with survival among penile cancer patients in the United States.

Methods: We utilized the Surveillance, Epidemiology, and End Results (SEER) Registry to identify patients with a primary diagnosis of penile malignancies from 2000 to 2019. Our primary outcome of interest was the hazard of death following a diagnosis of penile cancer. We utilized the Cox regression model to explore the association between race/ethnicity and median household income and how this influences survival among these patients. We adjusted for patients' characteristics, disease stage at presentation, and treatment modalities.

Result: Of the 6,520 penile cancer patients identified, 5,242 (80.4%) had primary malignancies. The distribution of patients was as follows: 64.1% non-Hispanic Whites, 8.9% non-Hispanic Blacks, 20.8% Hispanics, and 6.2% from other racial/ethnic groups. The median diagnosis age was 66 years (interquartile range: 56-74). Survival rates at 5, 10, and 15 years showed racial disparities: 76.4%, 72.5%, and 69.7% for non-Hispanic Whites; 70.6%, 64.1%, and 61.1% for non-Hispanic Blacks; and 70.5%, 67.4%, and 65.6% for Hispanics. Multivariate Cox regression revealed worst survival for Black (HR=1.40; 95% CI=1.08-1.81, p=0.01) and Hispanic patients (HR=1.24; 95% CI=1.01-1.52, p=0.04). No association was found between median household income and survival. Interaction analysis indicated that the poorest Black men had worse outcomes than the poorest Whites did (HR=2.08; 95% CI=1.27-3.41, p=0.003).

Conclusion: Survival rates for non-Hispanic Black and Hispanic patients are significantly lower than those for non-Hispanic Whites. Furthermore, survival is worse for low-income Black patients than their White counterparts in the same income bracket.

## Introduction

Disparities in health outcomes based on race/ethnicity and socioeconomic status are well-documented across many health conditions, necessitating continued exploration and action to address these entrenched inequalities [1-3]. This investigation is anchored in the context of penile cancer, a relatively rare malignancy in developed nations yet one marked by significant disparities in patient outcomes [4]. In 2023, an estimated 2,050 people in the United States will be diagnosed with penile cancer [5]. It is rare to find penile cancer in North America or Europe [5]. The disease accounts for less than 1% of all cancers diagnosed in men in the United States. Penile cancer is often associated with the human papillomavirus. It is more prevalent in Central Africa, Asia, and South America [5]. In 2020, 36,068 people were diagnosed with penile cancer worldwide [5,6].

Penile cancer represents an important area of study not merely due to its direct impact on those diagnosed but due to what it may reveal about the complex interplay between health outcomes and social determinants of health such as race/ethnicity and household median income [7-10]. These factors influence health outcomes across various diseases, yet the specific impact on penile cancer survival rates remains largely unexplored.

Race/ethnicity, often interacting with socioeconomic variables, has been identified as a significant predictor of health outcomes [11]. The factors leading to health disparities are multifaceted and complex, involving access to health care, lifestyle, the prevalence of risk factors, disease stage at diagnosis, and potential genetic susceptibilities [12]. Moreover, socioeconomic status, typically approximated by household median income, is recognized as another key determinant of health, encompassing elements such as education level, employment status, and living conditions, which can influence health outcomes both directly and indirectly [13].

Given this backdrop, our study endeavors to dissect the associations of race/ethnicity and household median income with survival rates among penile cancer patients in the United States. This examination will provide a comprehensive, focused investigation into these health disparities within the context of penile cancer, a disease for which such a detailed inquiry has been lacking.

## Materials and methods

The data utilized in this research was gathered from the Surveillance, Epidemiology, and End Results (SEER) Registry, spanning the years 2000 to 2019. The SEER database, a well-respected repository for American cancer data, provides reliable details on cancer prevalence and survival rates in the US and serves as an exclusive, comprehensive source of population-oriented data. This includes patient demographics, the primary tumor location, the tumor morphology, the cancer stage at the time of diagnosis, the treatment path followed, and patient updates on survival. The SEER program undergoes annual updates and is a valuable tool for a wide array of users, from clinicians and researchers to legislators, public health administrators, policymakers, and community organizations, for investigating the cancer load on the US population. Through the combined efforts of national data standards and several national committees, the SEER Program ensures the provision of accurate, high-quality data. This is achieved by comprehensive field edits designed to identify and rectify errors while checking for missing data and confirming codes.

Study population

The study focused on patients recorded in the SEER database who received a primary diagnosis of penile carcinoma during the period from January 2000 to December 2019. The only group excluded from this study were patients who were under 18 years of age at the time of diagnosis.

Patient characteristics and risk factors

This study accounted for several patient characteristics, including the stages of penile carcinoma as classified by the American Joint Committee on Cancer (AJCC), ranging from stages I to IV, and patients' racial/ethnic groups, which were identified as White (non-Hispanic White), Black (non-Hispanic Black), Hispanic, and Other. Patients' median household income was adjusted to align with 2019's inflation rates and then segmented into four quartiles. These quartiles consisted of Quartile I (earning less than $44,999), Quartile II ($45,000-$59,999), Quartile III ($60,000-$74,999), and Quartile IV (earning $75,000 and above).

In addition, we defined a geographical variable of urbanization. Non-Metro areas, not adjacent to any metropolitan region, were classified as 'Non-Metro.' We then further categorized metropolitan areas into three types: Metro I, with a population of 250,000 or less; Metro II, with a population ranging from 250,000 to 1,000,000; and Metro III, encompassing metropolitan areas with a population of 1 million or more.

We simplified marital status into two categories: married and unmarried. The unmarried category included individuals who were single, divorced, or widowed. Finally, the study considered the various treatment methods, encompassing surgery, chemotherapy, and radiotherapy.

Definition of study outcome

The primary outcome was the survival rate of patients post their diagnosis with penile carcinoma. We computed the hazard ratio (HR), or the time until death following the diagnosis, and excluded patients lacking comprehensive survival data from the analysis. Initially recorded in months, the survival variable was converted to years to facilitate the evaluation of patient survival at 5, 10, 15, and 20 years post-diagnosis.

Statistical analysis

Continuous variables in this study were presented as medians accompanied by their interquartile ranges (IQRs). These variables were then categorized according to the different racial/ethnic groups and evaluated using the Mann-Whitney U-test. Categorical variables, on the other hand, were depicted as frequencies and percentages and compared using the Chi-Square test. Survival data was generated, and the Kaplan-Meier curve was plotted, incorporating an at-risk table. The significance of this survival data was examined using the log-rank test.

The Cox regression model was employed to identify the relationship between patients' race/ethnicity, median household income, and overall cancer-specific mortality over time. Variables included in this model were patient age, disease stage at diagnosis, marital status, urbanization status, primary disease status, and the type of treatment received, whether surgery, chemotherapy, or radiotherapy.

The hazard ratio (HR), or the time to death, was calculated after adjusting for factors like patients' age, sex, race/ethnicity, insurance status, treatment modalities (surgery, chemotherapy, or radiation), and disease stage. We determined a two-tailed p-value less than 0.05 to be statistically significant. All statistical analyses were performed using Stata (Version 16, StataCorp, College Station, TX).

## Results

Table 1 displays patient characteristics and risk factors stratified by race/ethnicity. The total population had a median age of 66 years, with Hispanics being the youngest group at 59 years and Non-Hispanic Whites (NHWs) being the oldest at 68 years. This age distribution was statistically significant (p<0.001).

**Table 1:** Patients characteristics and risk factors stratified by race/ethnicity NHW, non-Hispanic White; NHB, non-Hispanic Black; IQR, interquartile range. p <0.001, statistically significant.

	Total Population	NHW	NHB	Hispanics	Others	p-Value
	(N=6,079)	(n=4,156)	(n=571)	(n=1,352)	(n=441)	
Median age (years) (IQR)	66 (56-74)	68 (59-76)	64 (54-72)	59 (48-70)	66 (56-74)	<0.001
Income						<0.001
Quartile I	727 (11.2%)	546 (13.1%)	112 (196%)	53 (3.9%)	16 (4.0%)	
Quartile II	1,578 (24.4%)	1,109 (26.7%)	165 (28.9%)	251 (18.6%)	53 (13.1%)	
Quartile III	2,469 (38.1%)	1,412 (34.0%)	205 (35.9%)	714 (52.9%)	138 (34.2%)	
Quartile IV	1,707 (26.3%)	1,089 (26.2%)	89 (15.6%)	332 (24.6%)	197 (48.8%)	
Stage						<0.001
Stage I	1,851 (54.3%)	1,222 (55.6%)	175 (55.9%)	333 (47.8%)	121 (59.6%)	
Stage II	669 (19.6%)	431 (19.6%)	48 (15.3%)	155 (22.3%)	35 (17.2%)	
Stage III	564 (16.5%)	348 (16.0%)	50 (16.0%)	135 (19.4%)	31 (15.3%)	
Stage IV	326 (9.6%)	197 (9.0%)	40 (12.8%)	73 (10.5%)	16 (7.9%)	
Metropolitan						<0.001
Non-Metro	1,073 (16.6%)	906 (21.8%)	73 (12.8%)	63 (4.7%)	31 (7.9%)	
Metro I	598 (9.2%)	438 (10.5%)	67 (11.7%)	78 (5.8%)	15 (3.8%)	
Metro II	1,369 (21.2%)	851 (20.5%)	121 (21.2%)	307 (22.7%)	90 (23.0%)	
Metro III	3,429 (53.0%)	1,961 (47.2%)	310 (54.3%)	902 (66.8%)	256 (65.3%)	
Surgery	5,711 (89.4%)	3,650 (89.1%)	490 (87.5%)	1,227 (91.6%)	344 (87.5%)	0.014
Chemotherapy	648 (10.0%)	357 (8.6%)	69 (12.1%)	185 (13.7%)	37 (9.2%)	<0.001
Radiation	535 (8.3%)	317 (7.7%)	48 (8.5%)	142 (10.7%)	28 (7.0%)	0.006
Marital status						<0.001
Married	3,561 (60.6%)	2,347 (62.4%)	227 (43.7%)	750 (59.7%)	237 (71.0%)	
Single	2,311 (39.4%)	1,414 (37.6%)	293 (56.4%)	507 (40.3%)	97 (29.0%)	
Primary cancers	5,206 (80.3%)	3,206 (77.1%)	464 (81.3%)	1,194 (88.3%)	342 (84.7%)	<0.001
Mortality	1,447 (22.5%)	901 (21.8%)	155 (27.5%)	321 ( 24.2%)	70 (17.6%)	0.001
5-year survival	75.30%	76.60%	71.10%	71.10%	81.00%	<0.001
10-year survival	71.10%	72.50%	64.20%	67.10%	78.10%	<0.001
15-year survival	68.20%	69.30%	61.30%	65.30%	75.10%	<0.001

Most patients fell into the third income quartile (38.1%). However, there were notable differences among racial/ethnic groups. Hispanics had the highest proportion in the third income quartile (52.9%), while non-Hispanic Blacks (NHBs) had the highest proportion in the second income quartile (28.9%). These income differences were statistically significant (p<0.001).

When looking at the disease stage at diagnosis, more than half of all patients were diagnosed at Stage I (54.3%). There was a significant variation across racial/ethnic groups (p<0.001). For instance, the Others group had the highest proportion diagnosed at Stage I (59.6%), whereas Hispanics had the lowest (47.8%).

Among the total population, 53.0% resided in the Metropolitan III areas. Hispanics had the highest proportion residing in Metropolitan III areas (66.8%), while NHWs had the least proportion (47.2%). These differences were statistically significant (p<0.001). A high percentage of the patients underwent surgery (89.4%), with Hispanics having the highest rate (91.6%) and NHB having the lowest rate (87.5%). This variation was statistically significant (p=0.014).

The use of chemotherapy was relatively low overall (10.0%), but higher in Hispanics (13.7%) and NHB (12.1%) compared to NHW (8.6%). The use of radiation was also relatively low (8.3%), with the highest usage seen in Hispanics (10.7%). Both chemotherapy and radiation usage varied significantly across racial/ethnic groups (p<0.001 and p=0.006, respectively).

About 60.6% of patients were married, but marital status varied significantly by race/ethnicity (p<0.001), with the highest proportion of married individuals in the Others group (71.0%) and the lowest in NHB (43.7%). Most patients had primary cancers (80.3%), with the highest percentage seen in Hispanics (88.3%). These variations were statistically significant (p<0.001). Mortality was 22.5% overall, with NHB having the highest rate (27.5%) and the Others group having the lowest (17.6%). This difference was statistically significant (p=0.001).

For survival rates, differences were noted across racial/ethnic groups. The 5-year survival rate was 75.3% overall, with the highest in the Others group (81.0%) and the lowest in NHB and Hispanics (both 71.1%). The 10-year survival rate was 71.1% overall, and the 15-year survival rate was 68.2%. Both survival rates showed significant differences by race/ethnicity (p<0.001).

This Cox regression analysis (Table 2) investigates the effects of various factors on the survival of patients diagnosed with penile cancer. The results of the Cox regression analysis are usually interpreted in terms of hazard ratios (HRs) and are presented below for each predictor.

Age: Each additional year of age is associated with a 1.8% increase in the hazard or risk of death (HR=1.01858, p<0.001). This means that older patients have a slightly higher risk of death.

Stage: Compared to stage I (reference category), patients in stages II, III, and IV had a higher risk of death. The risk is highest for stage IV patients (HR=7.03, p<0.001), followed by stage III (HR=2.95, p<0.001) and stage II (HR=2.13, p<0.001).

Race: Non-Hispanic Blacks (NHBs) and Hispanics have a significantly higher risk of death compared to non-Hispanic Whites (reference category), with HRs of 1.40 (p=0.011) and 1.24 (p=0.037), respectively. The category 'Other' race did not significantly differ from non-Hispanic Whites (HR=0.85, p=0.406).

Treatments: Patients who received chemotherapy had a 45.6% higher risk of death (HR=1.46, p=0.001). Those who received radiation also showed an elevated risk, but it was not statistically significant (HR=1.24, p=0.063). In contrast, surgery was associated with a 47.6% reduced risk of death (HR=0.52, p<0.001).

Median household income: There were no significant differences in risk of death between the income quartiles.

Metropolitan status: Living in different metropolitan areas was not associated with a significant difference in the risk of death.

Married: Married patients had a 17.5% lower risk of death (HR=0.83, p=0.020), indicating a potential protective effect of marriage on survival.

Primary cancers versus secondary malignancies: This variable did not significantly affect the risk of death (HR=1.06, p=0.597).

**Table 2:** Factors associated with survival among men with penile cancers (SEER 2000-2019) IQR, interquartile range; SEER, Surveillance, Epidemiology, and End Results. p shows statistical significance.

Hazard of Cancer-Specific Mortality	Hazard Ratio	95% Confidence Interval		p-Value
		Lower CI	Upper CI	
Age (years) (IQR)	1.019	1.011	1.026	<0.001
Income				
Quartile I	Reference			
Quartile II	0.887	0.699	1.175	0.404
Quartile III	0.909	0.657	1.261	0.569
Quartile IV	0.894	0.63	1.269	0.532
Stage				
Stage I	Reference			
Stage II	2.132	1.706	2.665	<0.001
Stage III	2.955	2.364	3.692	<0.001
Stage IV	7.033	5.519	8.962	<0.001
Metropolitan				
Non-Metro	Reference			
Metro I	0.809	0.576	1.139	0.226
Metro II	0.803	0.597	1.079	0.147
Metro III	0.761	0.564	1.028	0.075
Surgery	0.525	0.407	0.675	<0.001
Chemotherapy	1.457	1.168	1.816	0.001
Radiation	1.243	0.988	1.563	0.063
Married	0.825	0.703	0.969	0.02
Primary cancers	1.059	0.856	1.311	0.597

**Figure 1:**
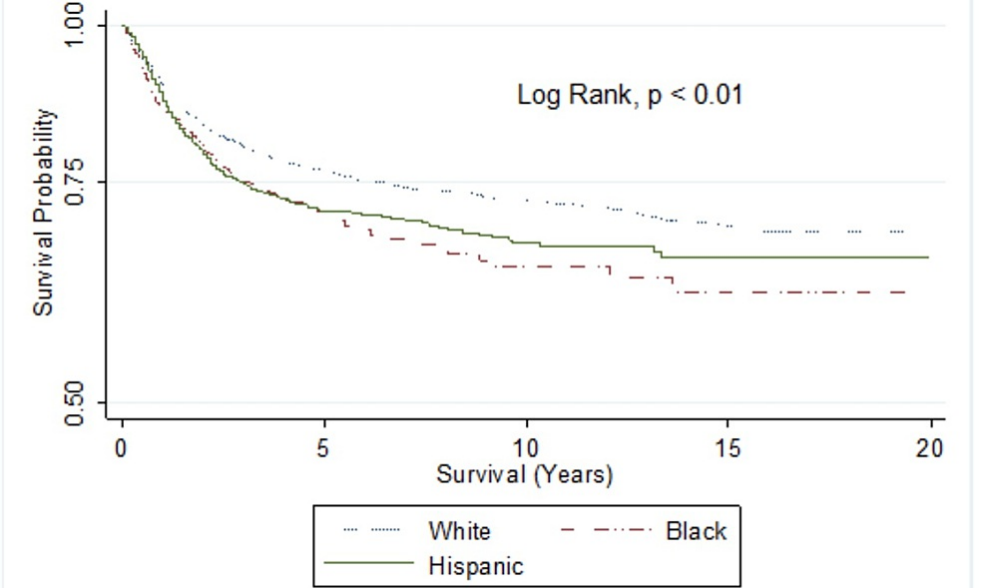
Kaplan-Meier survival estimates (Penile Carcinoma, SEER 2000-2019) SEER: Surveillance, Epidemiology, and End Results. p<0.01 shows a statistically significant difference in survival by race/ethnicity.

Figure 1 is a Kaplan-Meier graph that highlights the lower survival among non-Hispanic blacks compared to other races/ethnicities.

## Discussion

The findings of this study analyzed through a Cox regression model offer significant insights into various factors affecting survival rates in patients diagnosed with penile cancer.

Age

Consistent with previous studies [9,14], we identified age as a crucial factor, showing that each additional year of age corresponded with a 1.8% increase in mortality risk (HR=1.01858, p<0.001). While some research in this area, such as a study by Bourlon et al., reported that age ≥65 was not significantly associated with a higher incidence of death due to penile cancer [15], since our study utilized age as a continuous variable in the present study, we could not detect any changes in association across the different age categories and penile cancer mortality. Our findings emphasize that clinical decisions and prognostic assessments for penile cancer patients must consider age.

Cancer stage

Our study affirmed that the penile cancer stage significantly influences survival outcomes. This observation aligns with established literature [15-20]. We found the highest mortality risk in Stage IV patients (HR=7.03, p<0.001), followed by Stage III (HR=2.95, p<0.001) and Stage II (HR=2.13, p<0.001). This underlines the imperative of early diagnosis and prompt treatment initiation.

Regarding race as a variable, our study revealed a discernibly elevated mortality risk within the non-Hispanic Black population (HR=1.40, p=0.011), as well as among Hispanic individuals (HR=1.24, p=0.037) when compared with their non-Hispanic White counterparts. This finding could reflect a broader societal issue, mirroring the deeply entrenched racial disparities in the access to and quality of healthcare services received. Alternatively, it could indicate underlying biological variations among different racial groups, a topic previously explored in the literature [1,21]. However, the exact reasons behind this discrepancy necessitate further investigation to help develop effective strategies to mitigate this disparity in mortality risk.

Treatment type

The treatment modalities displayed differential outcomes [15,19,22-25]. While chemotherapy was associated with a higher mortality risk (HR=1.46, p=0.001), surgery was linked to a reduced risk (HR=0.52, p<0.001). This might reflect disease severity, as patients requiring chemotherapy may have more advanced or aggressive cancers.

Income and metropolitan status

Contrary to some studies that found links between socioeconomic status and cancer outcomes [26-28], our results revealed no significant differences in survival based on income quartiles or metropolitan status. This could suggest that in penile cancer, more direct factors such as comorbidities or healthcare access play a more critical role.

In congruence with prior studies [26,29-30], our study proposes that married patients seem to have a reduced probability of mortality risk, as indicated by a hazard ratio of 0.83 (p=0.020). This observed pattern could potentially suggest the influential role of psychosocial advantages linked with the institution of marriage on survival outcomes. The psychosocial benefits might include but are not limited to the emotional assistance a life partner provides and the resulting heightened likelihood of adhering to prescribed medical treatments. The interplay between these factors creates a complex, supportive environment that potentially enhances longevity.

In the context of our current investigation, we observed that the classification of the tumor as either a primary or secondary malignancy did not appear to have a notable impact on patient survival rates (HR=1.06, p=0.597). This finding diverges from previous research results, which have commonly suggested a significant correlation between the tumor's status, primary or secondary, and patient outcomes. Prior studies have typically associated better prognosis with whether the cancer is a primary or secondary malignancy [31]. Therefore, our findings introduce a potential reevaluation of this widely held notion and call for further exploration to validate this new perspective.

Policy implication

The findings of our study have profound policy implications and public health significance. Age, disease stage, race, and treatment type significantly influence survival rates in penile cancer, emphasizing the need for targeted public health strategies. Early detection programs should be reinforced, particularly in the minority and elderly populations at higher risk. Our findings also underscore the role of surgical intervention, which should be accessible to all, regardless of socioeconomic status. Moreover, the survival advantage in married individuals suggests the potential impact of psychosocial support in cancer management, which should be incorporated into holistic care approaches. In conclusion, applying our findings could lead to the development of health policies aiming at reducing disparities in penile cancer outcomes and improving overall patient survival.

Limitation

This study benefits from a robust dataset that allows us to explore the impact of several variables on the survival outcomes of penile cancer patients. Our use of Cox regression analysis is a significant strength as it effectively handles censoring and allows for the inclusion of multiple predictors. However, the analysis has its inherent limitations. For instance, the study's observational nature implies that we can only report associations, not causality. Moreover, the analysis did not include some potentially confounding variables, such as genetic predisposition, lifestyle factors, and comorbidities. The reliance on data from a single country may limit the generalizability of our findings globally, given that the risk factors and health infrastructure might differ substantially.

Despite these limitations, the study provides valuable insights into the survival of penile cancer patients. It underscores the impact of factors like age, race, disease stage, and type of treatment on survival outcomes. It also highlights areas requiring targeted intervention and the need for further research. Continued investigation into these factors, ideally in diverse populations, could provide a more comprehensive understanding of penile cancer survival, ultimately leading to improved patient care and outcomes.

## Conclusions

Our study provides important insights into the survival of penile cancer patients, demonstrating a significant influence of variables such as age, race, disease stage, and treatments on patient outcomes. The results suggest that as patients age, their risk of mortality increases, underscoring the need for a targeted approach in treating older patients. The higher mortality risk among patients in the advanced stages of the disease emphasizes the importance of early detection and intervention. Moreover, disparities in outcomes across different racial and ethnic groups call for a deeper exploration of socioeconomic, genetic, and systemic factors to address these disparities effectively.

Importantly, our findings illustrate the role of various treatments in patient survival. While chemotherapy was associated with a higher risk of death, surgery was found to reduce the risk, suggesting a possible direction for improving treatment protocols. However, the non-significant effect of income and metropolitan status on mortality risk means that other unmeasured factors might be at play. These findings highlight the need for a multi-pronged approach to managing penile cancer, focusing on early detection, individualized treatment plans, and addressing racial and ethnic disparities. Future research in diverse populations is essential to validate our findings and further improve patient outcomes.
